# Fast diffusion of domesticated maize to temperate zones

**DOI:** 10.1038/s41598-017-02125-0

**Published:** 2017-05-18

**Authors:** Xiaolong Li, Yinqiao Jian, Chuanxiao Xie, Jun Wu, Yunbi Xu, Cheng Zou

**Affiliations:** 1 National Key Facility for Crop Gene Resources and Genetic Improvement, Institute of Crop Science, Chinese Academy of Agricultural Sciences, Beijing, 100081 China; 2 International Maize and Wheat Improvement Center (CIMMYT), El Batán, 56130 Texcoco, Mexico; 3 Centre of Pear Engineering Technology Research, State Key Laboratory of Crop Genetics and Germplasm Enhancement, Nanjing Agricultural University, Nanjing, 210095 China

## Abstract

Adaptation to a temperate climate was a prerequisite for the spread of maize across a broad geographical range. To explicitly explore the demographic process underlying maize adaptation, we used a diffusion-based method to model the differentiation between temperate and tropical populations using the Non-Stiff Stalk group as a proxy for temperate maize. Based on multiple sequential Markovian coalescent approaches, we estimate that tropical and temperate maize diverged approximately 3‚000 to 5‚000 years ago and the population size shrank after the split. Using composite likelihood approaches, we identified a distinct tropical-temperate divergence event initiated 4‚958 years ago (95% confidence interval (CI): 4‚877–5‚039) from an ancestral population whose effective size was 24,162 (95% CI: 23,914–24,409). We found that continuous gene flow between tropical and temperate maize accompanied the differentiation of temperate maize. Long identical-by-descent tracts shared by tropical and temperate inbred lines have been identified, which might be the result of gene flow between tropical and temperate maize or artificial selection during domestication and crop improvement. Understanding the demographic history of maize diffusion not only provides evidence for population dynamics of maize, but will also assist the identification of regions under selection and the genetic basis of complex traits of agronomic importance.

## Introduction

Maize is a geographically widely distributed crop, grown from approximately 50°N to 45°S^1^. Although it originated from a single domestication event in southwestern Mexico^2^, a tropical zone, now more than 60% of maize is produced by countries that lie in the temperate zones^3^. The spread of maize to temperate areas required adaptation to changes in daily temperature, day length, soil type, and possible disease^4, 5^. Association studies and genome-wide scans for recent positive selection have been performed to predict genes involved in the adaptation to temperate zones and improved agronomic qualities^6–8^. However, limited knowledge of detailed demographic parameters increases the possibility that the genes identified in these studies are false positives^9–11^. We aimed, therefore, to explore the detailed demographic history of maize diffusion to temperate zones.

Considerable efforts have been made to estimate the time and the route of maize diffusion to temperate zones. Archaeological evidence supports the hypothesis that maize diffusion to temperate zones occurred through the Americas^12, 13^. Recent archaeological discoveries^14^ and ancient DNA sequencing^15^ have revealed that maize diffused to the southwestern United States (US) through the Mexico highlands and appeared in New Mexico or Arizona 4,100 years before present (BP). Based on the fixation index *Fst* estimation, Liu *et al*. augured that temperate-tropical divergence occurred 3‚400–6‚700 years BP, but this estimate was based only on genetic drift without accounting for changes in population size or gene flow between populations^8^. More complex models for maize diffusion have not been tested. In addition, although population-level transcriptomes have been generated for genome-wide association and pan-genome studies^16, 17^, few have been used to identify demographic events in maize history.

Various types of genetic information and algorithms have been used to infer population history. Coalescent simulation and approximate Bayesian computation (ABC)^18^ are widely used in inferring domestication bottlenecks and changes in population size in maize, rice, poplar and apple^19–21^. Using a coalescent model, maize was predicted to be domesticated 7500 years ago^22, 23^ through a single event^2^. With the development of high-throughput sequencing technology, another strategy that fits the site frequency spectrum (SFS) of single nucleotide polymorphisms (SNPs) with the proposed demographic model using a composite likelihood approach, named Diffusion Approximation for Demographic Inference (∂a∂i) has been widely applied to infer demographic history^24^. It has been used to estimate not only divergence events that occurred hundreds of thousands of years ago, such as between giant panda and polar bears^25^, but also domestication or effective population size changes in the time span of tens of thousands of years in soybean, common bean and rice^26–28^, and even to estimate bottlenecks within a time period of 100 years^29^. Recently, population genetic inference has been achieved by applying Markovian coalescent analysis (MSMC) to one or multiple genomes^30, 31^. MSMC can infer the changes in size of a single population or the timing and comparative population size of two populations that split from multiple phased haplotypes. Compared with the ABC method, ∂a∂i and MSMC are more efficient in handling high-throughput genomic data; therefore we chose these two methods to estimate the demographic history of the tropical and temperate split in this study. The biggest difference between the ∂a∂i and MSMC approaches is that the former requires a predefined model for demographic modeling. Under many scenarios with little precognition of demographic history, MSMC analysis will not only provide evidence for demographic change but also assist in constructing a more realistic model that can be used for other approaches that require a predefined model.

Iowa Stiff Stalk Synthetic (SS) and non-stiff stalk (NSS) are the two temperate maize populations that are the most widely used to produce hybrids^16, 32, 33^. SS and NSS have very similar origins and are an admixture of Northern Flint, Southern Dent and Tropical highland. In this study, we used NSS as a proxy for the temperate maize population for the following reasons: SS was generated from a very narrow genetic background in the 1930s^34^. Based on previous principal component analysis of the genetic diversity of different maize populations^16^, the genetic diversity of the SS population is small, thus it is inadequate to represent the generic diversity of temperate maize. If we combine NSS and SS together, an unbalanced sampling of these populations might introduce more artifacts in the simulation^35^.

To understand the demographic history of temperate and tropical maize, we used the tropical/subtropical (TS) and NSS populations to infer the demography of maize diffusion to temperate zones. We first employed MSMC, for which no specific demographic model is needed, to estimate the population statistics for different maize populations and determined that the split time between TS and NSS was approximately 3‚000 to 5‚000 years ago. We then estimated more detailed parameters including the strength and duration of the bottleneck during this split using the diffusion-approximation approach. The long identical-by-descent (IBD) tracts between populations were then identified. These long IBD tracts might be attributed to extensive gene flow/germplasm exchange between populations, or strong artificial selection during domestication or improvement and therefore might have contributed to maize adaptation to the temperate zone or to traits that meet human needs. The long IBD tracts identified in this study could be further examined and serve as targets for maize breeding.

## Results

### Characterization of maize populations

**Figure 1:**
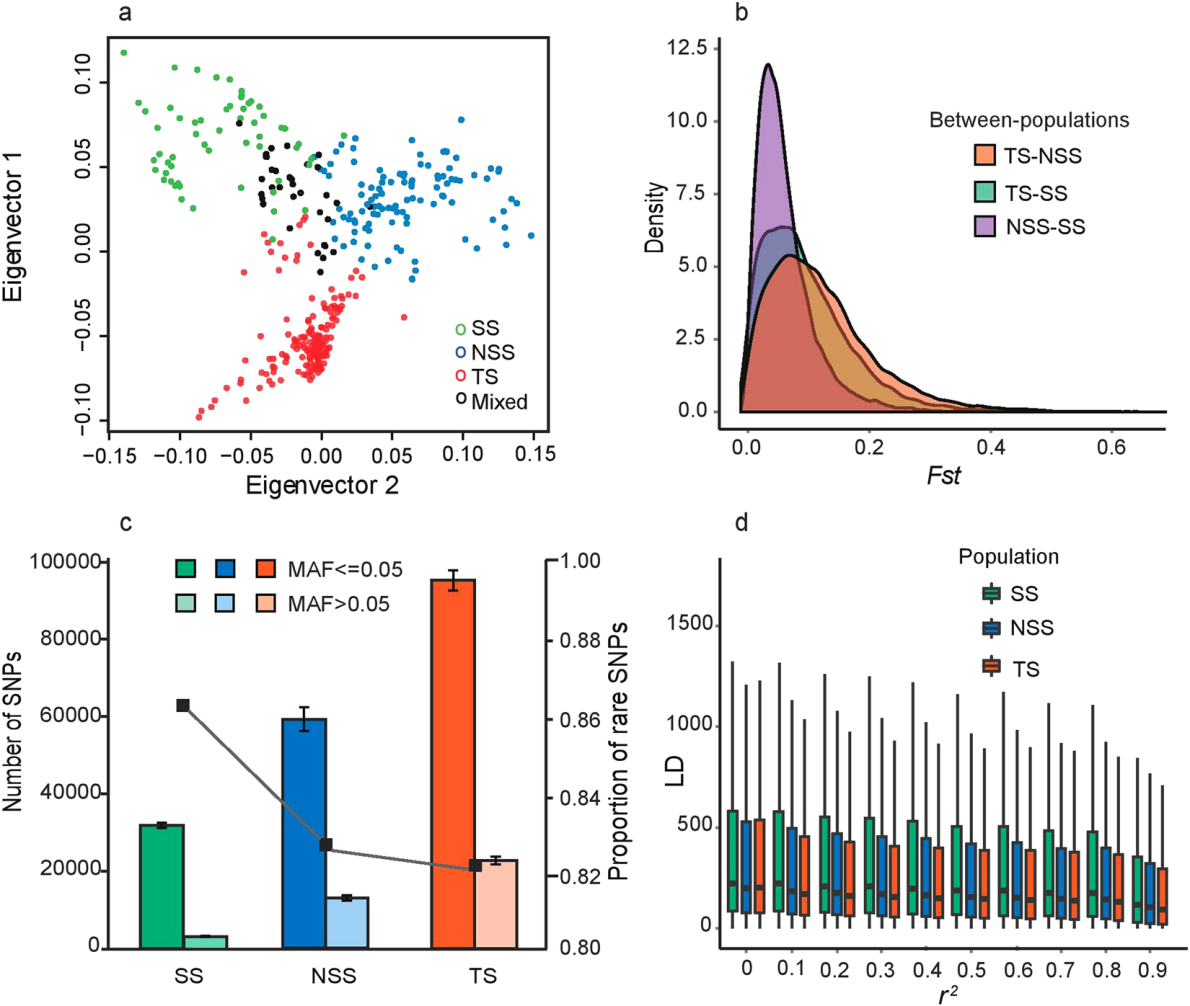
Population structure and characteristics of TS, SS and NSS populations. (**a**) Principal component (PC) analysis of all populations. The first two PCs are shown. Each individual is represented by one dot, colored according to population assignment. (**b**) Pairwise *Fst* for 1 Mbp sliding windows with a step size of 0.2 Mbp between populations. (**c**) The vertical bars indicate the total number of population-specific common (MAF > 0.05) and rare (MAF ≤ 0.05) SNPs, and the line plot shows the proportion of rare SNPs. (**d**) Boxplot charts of linkage disequilibrium (LD) decay distance estimated for categorized *r* ^2^ values.

Before examining the demographic history of maize populations, we characterized each population to help us to build more realistic demographic models. A total of 1.03 million high-quality single nucleotide polymorphisms (SNPs) generated from RNA sequencing of 368 maize inbred lines from Fu *et al*. were kindly provided by Dr. Fu^16^. From these data, Fu *et al*. determined the population structure with a Bayesian Markov Chain Monte Carlo (MCMC)^36^ approach using common SNPs with a low missing rate. We validated this population structure using a variational Bayesian framework with k = 3 and k = 4 implemented in fastSTUCTURE^37^. All 346 tested inbred lines are clearly structured into four subgroups including the two major US grain heterotic groups (SS and NSS), TS and mixed populations (Supplementary Fig. S1). It should be noted that the degree of admixture of different ancestries varies in individual lines. Only inbred lines with their main membership probability >0.60 were included in the ∂a∂i simulation described below. In addition, principal component analysis (PCA)^38^ of the genetic diversification of these populations indicated a clear separation of TS, NSS, SS and mixed populations. The first two principal components were used to visualize the relatedness between individuals and the four populations (Fig. 1a). During maize breeding, parental lines were generated within heterotic groups to ensure a heterotic effect in the hybrid. Thus, to avoid cryptic relatedness within samples, we eliminated the samples with a kinship greater than 0.5.

Next, we examined the divergence between SS, NSS and TS based on *Fst*, population-specific SNPs and linkage disequilibrium (LD). *Fst* is exponentially distributed and strongly peaked at 0.066 (NSS-TS) and 0.057 (SS-TS) (Fig. 1b). *Fst* between the temperate population, SS and NSS, is very small (peaked at 0.032). When randomly sampling 50 individuals from each population 10 times, we identified 20% of SNPs as being population-specific. The number of both common minor allele frequency (MAF > 0.05) and rare (MAF ≤ 0.05) SNPs are summarized in Fig. 1c. The number of population-specific SNPs in TS is 0.5 and two times larger than those in NSS and SS, respectively. The rare to common ratio of population-specific SNPs for SS is significantly greater than for NSS and TS. This difference might be attributed to the differences in NSS, SS and TS effective population size and/or weak purifying selection in the SS population^39^. As expected, we found that tropical maize exhibited faster LD decay (Fig. 1d) than the NSS and SS populations. The average distance over which LD decayed to a stable *r* ^2^ value (using 0.1 as a cutoff) is 170 bp in TS, 186 bp in NSS and 224 bp in SS in the genetic regions. We observed a faster LD decay than reported in previous studies^32^. This might be attributed to the fact that the marker density in Fu’s dataset is much higher than in the datasets used for these previous studies. In another study where high density markers (over 0.6 million SNPs) were genotyped for the lines in the USA national maize inbred seed bank. It was shown that the LD decayed to within from 100 bp to 500 bp for stiff stalk, non-stiff stalk and tropical population^33^.

### Inference of temperate and tropical maize divergence

**Figure 2:**
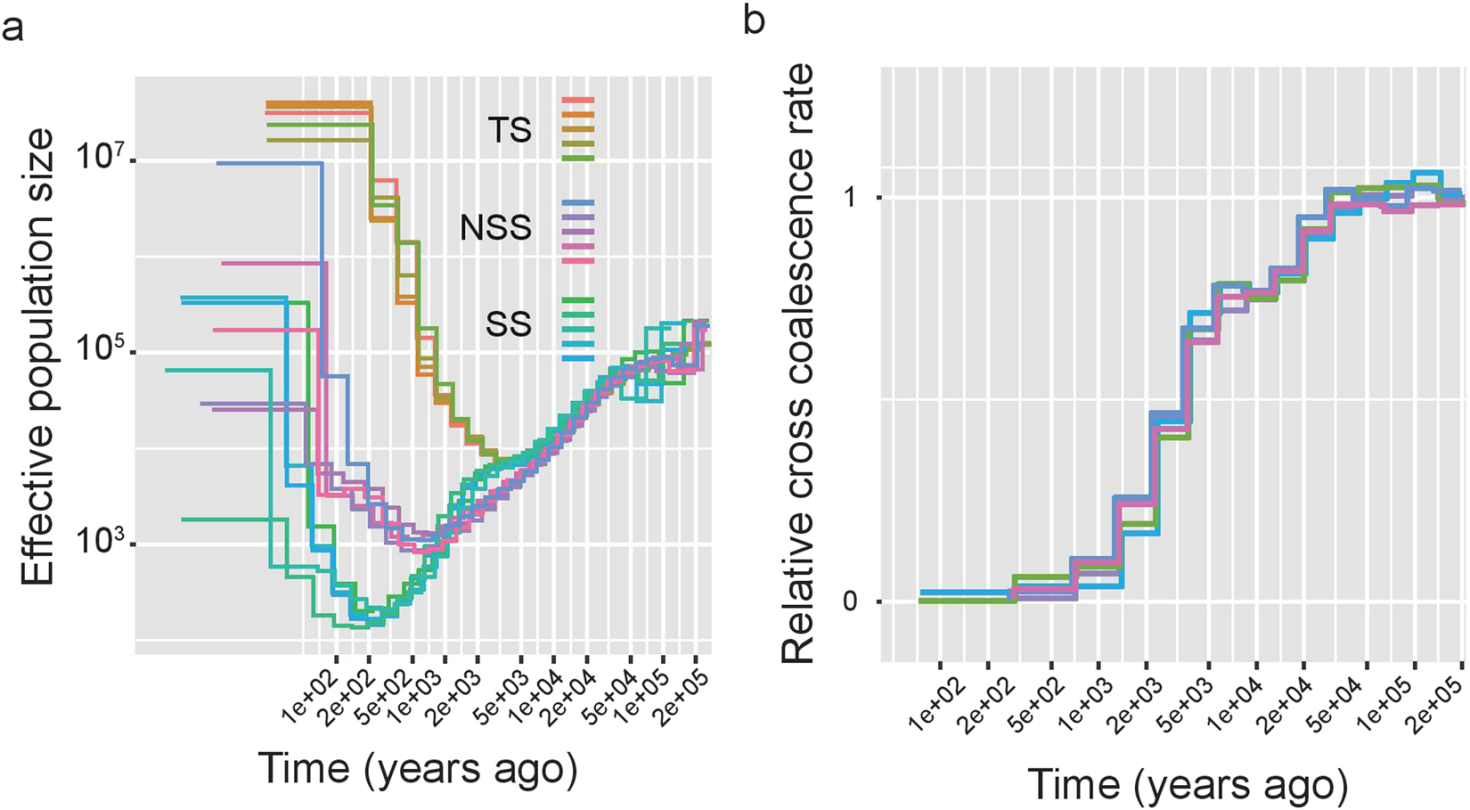
Population size and divergence inference using MSMC. (**a**) Population size estimation for the TS, NSS, and SS populations. For each population, five replicates were conducted with eight haplotypes. (**b**) The split time between TS and NSS was estimated from eight haplotypes (four haplotypes from each population). Five replicates were tested. To estimate the real split time and effective population size, we assumed a mutation rate of 3 × 10^−8^ and a generation time of one year.

To assess the pattern of tropical maize diffusion to temperate zones, we estimated the timing of divergence using two methods (MSMC and ∂a∂i)^24, 40^. The MSMC method does not require a predefined demographic model. Therefore we first employed MSMC to separately study the population size change for the TS, NSS and SS populations using the whole genome sequencing data for these inbred lines (details in Supplementary Table 1). The results from eight haplotypes are shown in Fig. 2a. After domestication, the population sizes of TS, NSS and SS sharply decreased. The TS population recovered from the bottleneck first followed by the NSS population. The SS population split from the NSS population in this century, so the bottleneck occurred very recently. We inferred the genetic split time between TS and NSS to be approximately 3‚000 to 5‚000 years ago (Fig. 2b).

We further inferred the demographic history using a diffusion-approximation approach^24^. To make sure the data are suitable for demographic inference, we revisited the original pipeline of SNP calling and processed the data to remove potential artifacts. First, we verified the accuracy of SNP calling in the previous pipeline. Although SNPs generated by the second generation sequencing platforms are error-prone, Fu’s SNP data set was already corrected using realSFS^41^, and the accuracy of SNP calling is high according to multiple validation methods^16^. Secondly, samples with hidden relatedness were removed from the analysis. Thirdly, we removed regions that have been predicted to be under strong natural or artificial selections during domestication or improvement^6^. Fourthly, only the 246,943 synonymous SNPs that have been predicted by SnpEff^42^ were included in the downstream analysis. Because the LD decays rapidly in genic regions (Fig. 1d), we randomly sampled SNPs and made sure that they were at least 2‚000 bp apart. We randomly performed the thinning approach ten times to ensure that SNPs thinning did not affect the model fitting. Our sample size was large, with more than 80 samples for each population. Thus, we projected our data into 60 haplotypes for each population. Both documented records and population diversity analysis indicated that the SS population was generated from a small group of founders in the 1930s^34, 43^, and the population size of SS is too small. Therefore, we used NSS as a proxy for the temperate maize population.

To test whether the allele frequency spectrum that we generated from inbred lines is suitable for the diffusion-approximation approach, we evaluated the fit of three different demographic models to the polymorphism data for the TS and NSS populations. We compared the neutral equilibrium model, the two-epoch model (corresponding to an instantaneous change in population size that occurred at a specific time), and the three-epoch model (a bottleneck that occurred at a specific time and lasted for a specific period of time). Because these three models are nested, we could perform the standard likelihood-ratio test and determin whether the difference in likelihood is significant. The log-likelihoods of various models are presented in Supplementary Table S2, and the difference between each model and the real data is reflected by Anscombe residuals provided by ∂a∂i (Supplementary Fig. 2). We found that both the two-epoch and three-epoch models significantly outperformed the neutral equilibrium model (P-value < 1.0 × 10^−6^, df (degrees of freedom) = 2) for the TS and NSS populations; however, the difference between the two-epoch and three-epoch models is not significant. When parameters are converted into physical units, the best fitting model indicates that the effective population shrank 4‚134 and 3‚387 years ago in the TS and NSS populations, respectively.

**Figure 3:**
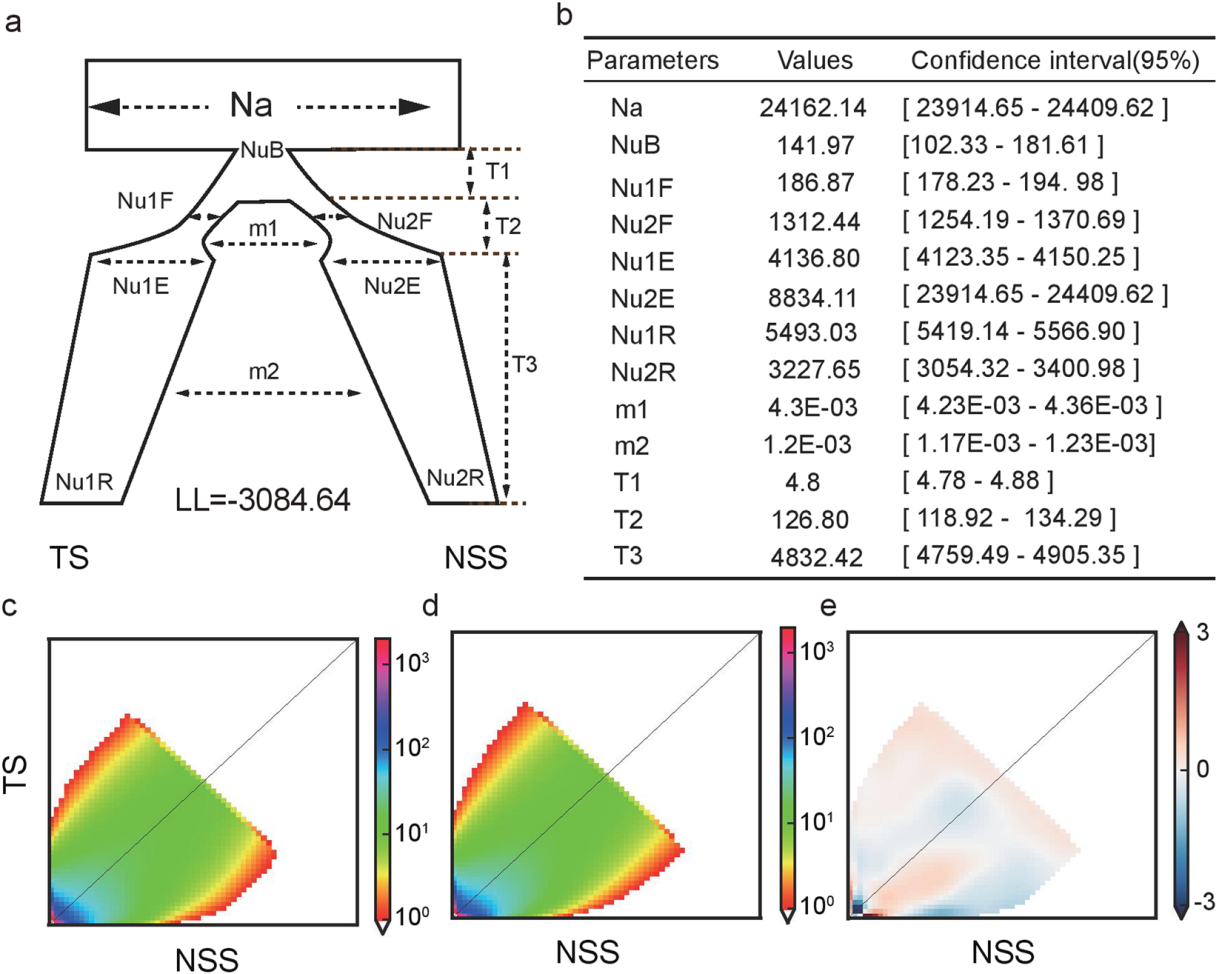
Population divergence estimated by ∂a∂i. (**a**) The demographic model allows for two successive bottlenecks caused by domestication and founder effects of temperate population formation. The duration of the domestication bottle neck (*T1*), population split and recovery time (*T2*), and population divergence time (*T3*) were estimated. The population size at each time point was also estimated. The migration rates are expressed as *M*ij, where *M*ij/2*Na* = *m*ij, the proportion of individuals in population j who are new migrants (*M*) from population i every generation, and mij is the migration parameter estimated in ∂a∂i. (**b**) Best-fit parameters inferred by ∂a∂i simulations. The transformation to physical units is described in the Materials and Methods. (**c**) The joint allele frequency spectrum of the observed data. (**d**) The joint allele frequency spectrum built from simulated data according to the best model. (**e**) The Anscombe residuals between the simulated and observed data.

Because both archaeological and molecular data indicate a single domestication of cultivated maize, we only considered the models where NSS directly split from domesticated tropical maize. To simplify the question, we first compared demographic models simulating the split between the TS and NSS populations without considering the change in population size before or after the split (Supplementary Fig. 3A). In the simplest model, an instantaneous change in population size is allowed in both populations at the onset of the split. We inferred the parameters *Na* (the effective size of the ancestral tropical maize population), *nu1* (the size of the TS population immediately after the split), *nu2* (the size of the NSS population immediately after the split), *T* (time in the past at which the split began) and *m* (migration rate between populations). Based on the results from models for one population, we found that population downsizing happened earlier for TS than for NSS, which might suggest the bottleneck effect of domestication; therefore, we constructed a second model that allows a population size change before the split (Supplementary Fig. 3B). In the third model, a population size change in NSS is specified after the split (Supplementary Fig. 3C). In the fourth model, a bottleneck with an exponential increase in size before the split is added (Supplementary Fig. 3D). Our simulations showed that the third model and the fourth models have the minimum Akaike information criterion (AIC) values^44^, and the likelihood ratio tests considering models 3 or 4 as a null hypothesis and models 1 or 2 as alternative hypotheses are both significant (P-value < 1.0 × 10^−13^, df = 2) (Fig. 3).

Based on the MSMC result, the effective population size of TS, NSS, and SS gradually shrank after the split. In addition, it is unlikely that there was instantaneous population downsizing during the divergence of maize. Therefore, we constructed a fifth model that allows a linear downsizing of the subpopulations (Fig. 3a). The new model includes a double bottleneck, which characterizes the domestication process and population split separately, population downsizing after the split, and gene flow between TS and NSS. The likelihood ratio test shows that the fifth model is the best among the five models we tested. Model parameters and confidence intervals are displayed in Fig. 3b. Our models indicate that the ancestral population size of maize was approximately 24‚162 (95% CI (confidence intervals) [23‚914–24‚409]) and that the TS and NSS populations split 4‚958 years ago (95% CI [4‚877–5‚039]). The bottleneck before the split was very severe (142 individuals) and of short duration (5 generations). The ancestral temperate founder population is estimated to be 1‚312 individuals. Divergence persisted for approximately 126 generations. After that, both tropical and temperate population underwent gradually shrinking for 4‚832 years, with their population sizes decreasing by 25% and 65%, respectively. Extensive gene flow is predicted at both the split-recovery stage and the shrinking stage, which is 4.3 × 10^−3^ and 1.2 × 10^−3^ migrants per generation, respectively. To further validate the strength and the pattern of gene flows between TS and NSS, we analyzed IBD sharing among populations.

### Gene flow and potential regions under selection during maize diffusion

**Figure 4:**
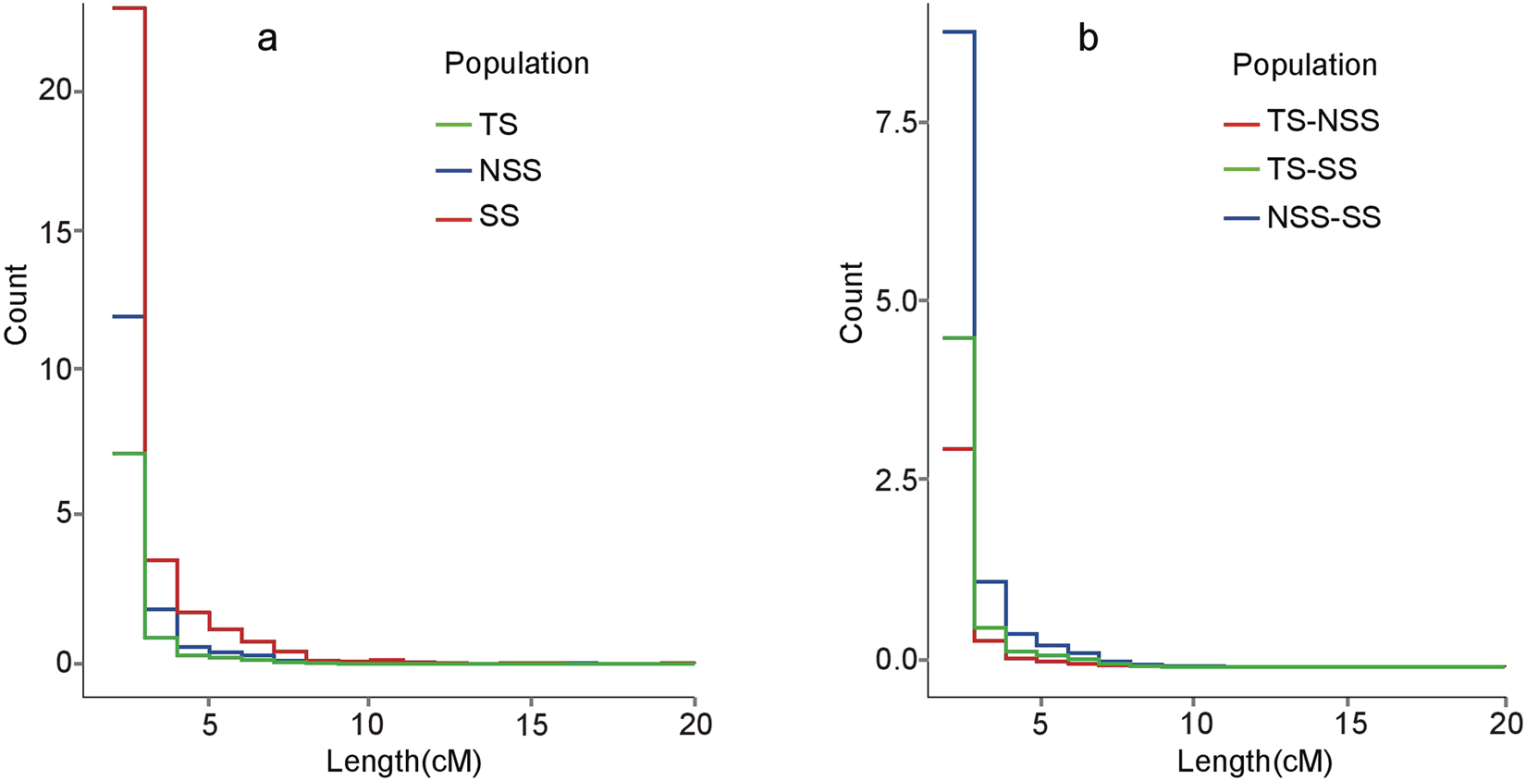
The length distribution of IBD tracts within populations (**a**) and across populations (**b**). The number of IBD tracts are normalized by the number of individuals (in **a**) or individual pairs (in **b**).

Extensive gene flow between TS and NSS has been predicted by ∂a∂i. IBD tracts, the DNA segments shared between individuals, are informative of demographic and evolutionary events in the population. Although gene flow could introduce IBD between populations, it is worth noting that strong natural or artificial selection also give rise to IBD tracts that are commonly shared between lines. To further explore the intensity of the gene flow and potential regions under selection, we evaluated IBD sharing within and between populations. IBD segments smaller than 1 cM were eliminated because they are more likely to be affected by background LD. The length distributions of IBD segments within and between populations are shown in Fig. 4. As the length of IBD segments increases, the frequency of IBDs segments decreases dramatically. This is likely due to the elevated recombination rate in maize^45^ and breakdown of IBDs tracts when desirable loci were selected by breeders. The TS population exhibits less within-population IBD sharing compared to the other two populations (Fig. 3a). We also found that the IBD sharing between TS and SS is more extensive than that between TS and NSS. On average, a single tropical line shares 6.66 cM segments with an NSS line but shares 10.66 cM segments with an SS line.

**Figure 5:**
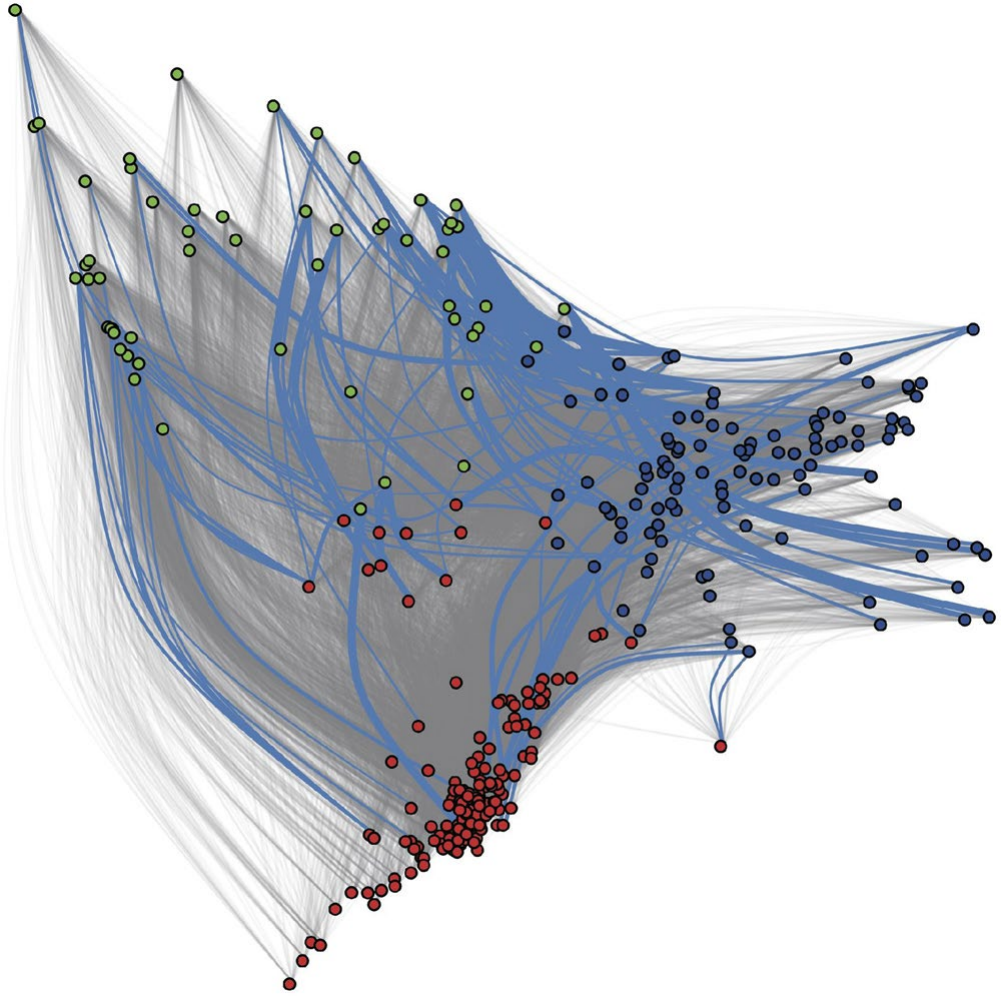
IBD sharing between the TS, NSS and SS populations. Red dots represent TS inbred lines, blue dots represent NSS inbred lines, and green dots indicate SS inbred lines. Edge width indicates the mean length of IBD tracts shared between one TS sample and one NSS/SS sample. The grey line connecting two samples indicatesthat the median length of IBD tracts is less than 7 cM (top 1%). A blue line connecting two samples indicates that the median length of IBD tracts is greater than 7 cM.

To determined if any specific tropical lines have been intensively used as donors of germplasm introgression, for each tropical line we analysed the IBD segments that are shared with NSS or SS individuals (Supplementary Table S3). We found that the total length of shared IBD between TS lines and NSS/SS lines are in disequilibrium. For instance, the TS lines ZHONG69, CIMBL42, CIMBL143 and CIMBL141 share large segments of IBD with NSS lines. These lines were classified as a mixed group in a previous study^16^, although more than 50% of their genomes are of tropical ancestry based on our population structure analysis. Therefore, to eliminate the possibility that IBD only exists between close relatives or mixed inbred lines, we plotted the amount of IBD sharing against genetic similarity, which is represented by the first two components of PCA analysis shown in Fig. 1a (Fig. 5). Based on the random expectation, the chance of two 19th descendants sharing a 7 cM IBD block is very slim; therefore, we used 7 cM as a threshold to define small and long IBD blocks. We plotted short IBD blocks (<7 cM), which are universal among all inbred lines (grey lines in Fig. 5) and long IBD blocks (>7 cM), which tend to be concentrated in a few lines. Of particular interest is the finding that IBD sharing not only occurs between closely related lines but also between distantly related lines.

**Figure 6:**
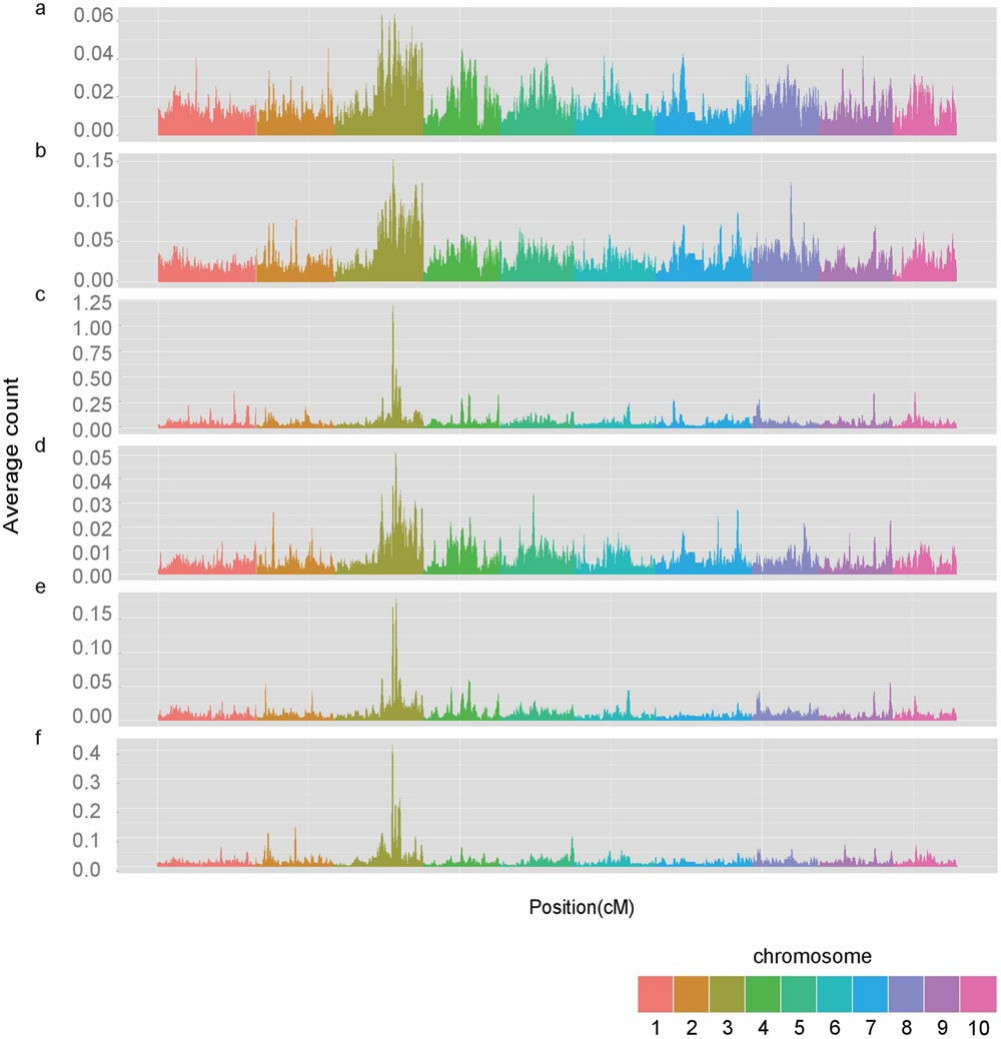
Hotspots of IBD sharing within populations and across populations. The occurrence of IBD is averaged by sample number and is indicated on the y axis. IBD occurrence (**a**) within the TS population; (**b**) within the NSS population; (**c**) within the SS population; (**d**) between the TS and NSS populations; (**e**) between the TS and SS populations; (**f**) between the NSS and SS populations.

The distribution of IBD blocks across the genome is also imbalanced. Regions with extensive IBD sharing are indicative of regions under positive selection during domestication or improvement. The average frequency of IBD segments spanning each 10 cM window are displayed in Fig. 6. Of the IBD regions with the highest frequency (top 5%), 10.7% and 9.7% of IBD overlap with regions that were previously predicted to be under selection during maize domestication and improvement^6^, respectively. This overlap is greater than expected by chance (P < 0.03, based on a random permutation). A region in bin 3.08 on chromosome 3 is commonly shared within all of the populations and between all populations and, which might be a target of strong selection. We also found a peak located in bin 8.06 that is enriched in IBD tracts within the NSS population. Genes located in these two peaks were annotated using slim gene ontology and pfam HMM model (Supplementary Table S4). Using the same SNP dataset, Yan’s group also detected strong selection in bin 3.08 and bin 8.06 during tropical and temperate divergence^8^, and nine genes in the regions they identified are also found in the IBD tracts we identified in our study. Moreover, bin 8.06 has been detected as a selected region in comparisons of historical germplasm of North American maize^46^.

## Discussion

### Fast diffusion of tropical maize to temperate zones

In this study, we reconstructed the demographic history of the tropical-temperate split using the MSMC and the ∂a∂i approach. Our results indicated that the diffusion of domesticated maize to temperate zone was fast and might have occurred immediately after the domestication. Based on MSMC, the divergence time between the TS and NSS populations is predicted to be approximately 3‚000 to 5‚000 years. This is consistent with a recent estimate of 3‚﻿400 to 6‚700 years obtained using *Fst* ^8^. However, the confidence interval (95% CI: 4‚877–5‚039) from our ∂a∂i analysis is much narrower than the *Fst* estimation, which suggests less uncertainty. In addition, this estimation is consistent with the archaeological records^14, 15^. A recent archaeological study suggested that maize spread to Peru from 5,000 to 6,200 years ago^47, 48^. The corn collected from five sites in Arizona and New Mexico predates 2,000 B.C., which indicates that maize diffusion to the US Southwest can be traced back to 4,000 years ago^14^. Considering previous estimations and the archaeological record, our simulation provides an slightly earlier and more accurate estimate of the split date of tropical and temperate maize that is in keeping with the expectation.

Our result also indicates that the time between domestication and the split was short. This estimation is consistent with a previous experiment in which researchers tried to adapt tropical maize germplasm to a temperate environment. After six cycles of selection in Urbana, IL (located at 40°6′35″N 88°12′15″W), flowering time in a photoperiod insensitive maize collection with 13 tropical populations was reduced by 14 days^49^. This experiment can be treated as an example of maize diffusion from tropical to temperate zones and indicates that under strong artificial selection, tropical maize can adapt to the temperate environment in a very short period of time. This provides support for our hypothesis that there was fast diffusion of maize from tropical to temperate zones.

After recovering from the domestication bottleneck, the temperate population split from the tropical population with a founder population of 1‚312 individuals (5% of the ancestral population). Compared with the domestication bottleneck (0.5% of the ancestral population in our simulation), the temperate bottleneck was less severe and persisted a comparatively longer time. A moderate bottleneck also occurred when maize was introduced from the US to Europe; the diversity of European maize decreased 25% compared to that of American maize. Given that our estimations were based on simulations with the genetic variance between tropical and temperate inbred lines, these lines might not be the most optimal population to conduct this study. More archaeological studies and population analyses with local landraces or ancient maize from archaeological sites will help to clarify the demographic history of the tropical-temperate split.

### Inferring a demographic model for the tropical-temperate split event

Using inbred line populations to infer demographic history seems to violate the assumption of panmixia in population genetic research. However, maize was a widely openly pollinated species for a long time before modern plant breeding started early in the last century, so the departures from randomness in mating might be small enough to be negligible. Therefore, with high-density genomic markers and many sequenced samples in hand, using the breeding populations as a proxy for inferring demographic models of the tropical-temperate split is a worthy endeavor. Our results are consistent with previous studies in several key parameters. Our best fitting model inferred the effective population size of the ancestral populations to be around 24,000. This estimation is close to an estimation made using microsatellites. The authors of this study argued that the lower and upper bounds of the effective population size for maize is 13,100 and 33,000, respectively^50^. However, an estimation as large as 200,000^51^ has also been made, which might be attributed to the higher population recombination frequency the author used. A recent estimation of the ancestral effective population size using teosinte genomes is approximately 123,000 (http://www.nature.com/articles/nplants201684)^8^. This difference between this estimate and ours might be attributed to the fact that we used inbred lines to estimate the ancestral population size, which might lead to an underestimation. Our simulation strongly supports a severe bottleneck prior to the TS-NSS split, which is coincident with the known bottleneck in maize domestication (approximately 5% of ancestral population)^8^. In addition, our best fitting model indicated a prompt recovery of the ancestral teosinte population; at the onset of the split, the total population size of TS and NSS was 10,746, which indicates that in the maize population we studied, 50% of the ancestral population recovered soon after domestication.

Although the diffusion-based approach has been widely used to inferring demographic history for many species and for many scenarios, there are still some limitations. First, an accurate SFS is crucial to make precise demographic inferences. Multiple algorithms have been proposed to correct the SFS^52, 53^, and in this study we used a Bayesian approach, RealSFS^52^ to correct the dataset^16^. Second, many factors influence the conversion of model parameters solved by ∂a∂i to physical units. The ancestral population size is proportional to the effective length sequenced and the mutation rate. The effective length is harder to determine in second generation sequencing than in Sanger sequencing because of the variation in sequencing coverage. In addition, the estimation of the nucleotide substitution rate is under debate. Based on a study of the *tb1* intergenic region in maize and teosinte using the Bayesian approach, the nucleotide substitution rate has been estimated to be approximately 3.310 × 10^−8^ (95% CI is 2.0–5.0) per bp per generation^54^. In a recent study where a genome-wide pedigree-base was estimated, the nucleotide substitution rate of the genetic region was 4.794 × 10^−8^ per bp per generation^55^. A reliable estimation of the substitution rate is the basis for accurate demographic inference; therefore, future studies needs to be conducted to obtain a more accurate estimation of the substitution rate. Although the inaccuracy in substitution rate will affect the estimation of absolute divergence time of tropical and temperate maize, the interval between the domestication bottleneck and tropical-temperate split event is short in our simulation, which is less affected by the substitution rate. Therefore, we concluded that the diffusion of domesticated maize to temperate zone after domestication was fast.

### Demography and population characters

Population structure and many population divergence indices are strongly affected by changes in demographic history, such as expansion, bottlenecks and gene flow. In this study, we found that many population characteristics are consistent with the demographic models we drew from joint SFS. For example, in our simulation we found that the current effective population size of TS is larger than that of NSS. In this study, we found that there are more population-specific SNPs, faster LD decay, and a lower level of IBD in TS than in NSS. The difference in effective population size could be one, but not the only explanation for all of these observations. We also observed a higher ratio of population-specific SNPs in SS, which might be attributed to SS population expansion. The initial population of size of SS was less than 20 parental lines. But many inbred lines have been developed using several elite lines. For example, there are more than 50 inbreds lines that share more than 97% of their genomes with B73^33^. We observed a moderate *Fst* between TS and NSS. This estimate is consistent with several previous studies that reported an average *Fst* of 0.06 between tropical, SS and NSS populations^33^. The differentiation between tropical and temperate maize is moderate and is smaller than between tropical and temperate cultivated rice^56^ (0.50 between indica and japonica) and greater than between tropical and temperate cultivated soybean (0.005)^26^.

### Breeding process and demography

The demographic history of cultivated plants reflects a process of meeting human needs and adapting to new environments. Extended IBD/haplotypes have been identified in many populations and seem to be related to genes involved in stress responses and human directional selections^8^. Another resource of large IBD tracts might due to the germplasm introduced by breeders. For example, in the 1980s, breeders introduced some tropical germplasm to SS, which has been used as the female parent in hybrid production. In this study, we have identified multiple long identical-by-descent tracts shared by tropical and temperate inbred lines, some of which are consistent with previously detected regions under selection during maize breeding and some of which might be attributed to the gene flow between tropical and temperate maize. In summary, the demography of maize is largely affected by the breeding process.

## Methods

### Data processing

A total of 1.03 million high-quality SNPs generated from RNA sequencing of 368 maize inbred lines were adopted from Fu’s study^16^. To avoid the strong influence of SNP clusters in the population structure and sample relatedness analyses, LD-based SNP pruning was performed using the bioconducter package SNPRelate with an *r* ^2^ threshold of 0.2 and a window size of 500‚000 base pairs^57^. To calculate the sample relatedness, IBD estimation was conducted using the method of moments implemented in SNPRelate. When kinship between two samples was greater than 0.5, one of the samples was randomly removed from the analysis. A total of 22 samples were removed from the analysis.

### Population structure analysis

The population structure was investigated using both a non-parametric approach using a variational Bayesian framework (fastSTRUCTURE)^37^, and principal component analysis implemented in SNPRelate using LD-pruned SNPs with minor allele frequency (MAF) > 0.05. Different numbers of ancestral clusters (k = 2 through 5) were tested successively with the default convergence criterion ten times. The results from different replicate runs were integrated using the CLUMPP program with the full search algorithm^58^.

### Population divergence and linkage disequilibrium analysis

Genomic divergence between different populations and pairwise nucleotide diversity within a population were calculated using VCFtools version 0.1.12.0 via the Weir and Cockerham estimator^59^ of *Fst* ^60^ with a window size of 1 Mb. Windows with more than 30 segregating sites were analysed. Population-specific SNPs were extracted by VCFtools. Because the population size affects the number of population-specific SNPs, we randomly sampled 50 individuals from each population. The result from 10 independent runs were summarised. The *r* ^2^ of ten adjacent SNPs were calculated using PLINK 1.07^61^, and a boxplot of different *r* ^2^ bins in different populations was generated by ggplot2 in R. Population-specific SNPs were selected by VCFtools.

### Demographic inference with MSMC

Hapmap 3 has released the genotypes of 916 diverse inbred lines of maize. We first downloaded the whole dataset from maizeGDB (http://www.maizegdb.org/diversity). Samples with TS, NSS and SS genetic ancestry greater than 99% were selected as candidate haplotypes. We made a fake dipoid by randomly joining two haplotypes in the same population. The MSMC analysis were conducted using the updated version (https://github.com/stschiff/msmc2) using pattern parameter 20*1.

### Demographic inference with ∂a∂i

A total of 246,943 synonymous SNPs were identified by SnpEff. Regions predicted to be under positive selection during domestication and improvement were eliminated from the analysis^6^. The number of SNPs was further reduced using the thin option in PLINK^61^, leaving 13.6% of these SNPs located at least 2 kb apart. Joint allele frequency spectra between TS and NSS were derived from this dataset. The data were then hypergeometrically projected to 60 samples to eliminate the influence of missing data. Demographic modeling was performed with ∂a∂i version 1.6.3^24^. For each model, we performed the simulation with an exhaustive search of the initial parameter to reduce artifacts introduced by improper choice of initial parameters. For each parameter, we used at least two starting parameters one magnitude apart. We investigated and compared different demographic models based on the relative log-likelihoods of the models given the observed site frequency spectrum. To compare the models with different numbers of parameters, we calculated the Akaike information criterion (AIC)^44^ for each model, and the model with the minimum AIC value was preferred in our analysis. Confidence intervals were derived based on the simulation results from the bootstrap method. The reference population size *Na* was calculated using the equation *theta* = *4* × *Na* × *μ* × *L*, where *μ* is the mutation rate and *L* is the effective sequence length. In our analysis, the total length of *L* was the sum of the length of exons containing at least one SNP. This number was then multiplied by 13.6% to obtain the approximate effective length after SNP thinning (*L* = 3.8 Mbp), because we filtered 86.4% of SNPs in thinning. Based on a recent study of genome-wide pedigree-based estimation, the average nucleotide substitution rate per gene is 4.794 × 10^−8^ ^55^, and the nonsynonymous-to-synonymous ratio is 1.14, therefore, we used *μ* = 2.230 × 10^−8^.

### Identification of IBD segments

The kinships between samples was estimated by identical-by-state (IBS) pairwise identities using the snpgdsIBS function in the SNPRelate package in R^62^. Samples with kinships greater than 0.5 were removed from the downstream analysis. For each pair of inbred lines, IBD was calculated by fastIBD with the default threshold 1.0 × 10^−8^ ^63^. The consensus IBDs, generated by 10 independent runs, were used for downstream analysis. The physical positions of SNPs were transformed into genetic positions by a linear interpolation based on a randomly chosen population, CFD03^64^. IBD segments smaller than 1 cM were eliminated, because they tend to be affected by background LD^65–69^. To detect the hotspots of IBD sharing, we calculated the average occurrence of IBD segments between two populations for 10 cM non-overlapping windows by dividing the number of IBD segments spanning the window by the number of all possible pairs. Genes within an IBDs sharing hotspot were extracted and annotated by slim plant ontology and Pfam domain prediction.

## Electronic supplementary material

Supplementary information

Dataset 1

Dataset 2

Dataset 3

Dataset 4

Dataset 5
