# Supplementary material for: Fast diffusion of domesticated maize to temperate zones
Source: Sci Rep. 2017 May 18;7:2077. doi: 10.1038/s41598-017-02125-0 (PMC5437101; doi:10.1038/s41598-017-02125-0)
Supplement: Supplementary file 1 — Supplementary information [file 41598_2017_2125_MOESM1_ESM.pdf]

Fast diffusion of domesticated maize to temperate zones

Xiaolong Li[1,3], Yinqiao Jian[1], Chuanxiao Xie[1], Jun Wu[3], Yunbi Xu[1,2,*], and Cheng Zou[1,*]

[1]National Key Facility for Crop Gene Resources and Genetic Improvement, Institute of Crop Science, Chinese Academy of Agricultural Sciences, Beijing 100081, China

[2]International Maize and Wheat Improvement Center (CIMMYT), EI Batán 56130, Texcoco, Mexico

[3]Centre of Pear Engineering Technology Research, State Key Laboratory of Crop Genetics and Germplasm Enhancement, Nanjing Agricultural University, Nanjing 210095, China

[*]Corresponding author: zoucheng@caas.cn, y.xu@CGIAR.ORG

Supplementary Information

Supplementary table S1

Whole genome sequencing data for the TS, NSS and SS inbred lines used for ∂a∂i and MSMC.

Supplementary table S2

Performance of three different demographic models using data from the TS and NSS populations.

Supplementary table S3

IBD sharing distribution between individuals.

Supplementary fig. S1

Results from NSS (non-stiff-stalk), SS (Stiff Stalk) and TS (tropical/subtropical populations) population structure analysis. Results for K=3 and K=4 are shown. Each individual's genome is represented by a vertical bar composed of colored sections, where each section represents the proportion of an individual's ancestry derived from one of the K ancestral populations. Individuals are arrayed horizontally and grouped by groups as indicated.

Supplementary fig. S2

1D model-data comparison plots. In the top panel, the model is plotted in red and the data in blue. In the bottom panel, the residuals between the model and data are plotted. The top plot shows the model and data frequency spectra, whereas the bottom plots the residuals between the model and data. A, Standard neutral model (snm) for TS; B, two epoch model for TS; C, three epoch model for TS; D, Standard neutral model for NSS; E, two epoch model for NSS; F, three epoch model for NSS; G, Standard neutral model for SS; H, two epoch model for SS; I, three epoch model for SS.

Supplementary fig. S3

Comparison of four demographic models with different migration patterns using ∂a∂i. (A) None gene flow is simulated. (B) One-way gene flow is simulated (C) Symmetric gene flow is simulated (D) Asymmetric gene flow is simulated. The log-likelihood and AIC value were presented for each model.
