# Supplementary material for: Fast diffusion of domesticated maize to temperate zones
Source: Sci Rep. 2017 May 18;7:2077. doi: 10.1038/s41598-017-02125-0 (PMC5437101; doi:10.1038/s41598-017-02125-0)
Supplement: Supplementary file 3 — Dataset 2 [file 41598_2017_2125_MOESM3_ESM.doc]

Table S2: Performance of three different demographic models using data from TS and NSS populations.

|  | | | | | Snm(TS) |  | | |
| --- | --- | --- | --- | --- | --- | --- | --- | --- |
| Theta | Na | |  | |  |  | Log likelihood | AIC |
| 4934.405 | 1 | |  | |  |  | -615.281 | 1230.562 |
|  | 12023 | |  | |  |  |  |  |
|  |  | |  | | Snm(NSS) |  |  |  |
| Theta | Na | |  | |  |  | Log likelihood | AIC |
| 4671.933 | 1 | |  | |  |  | -1694.418 | 3388.836 |
|  | 11384 | |  | |  |  |  |  |
|  |  | |  | | Two_epoch(TS) |  |  |  |
| Theta | Na | | nu | | T |  | Log likelihood | AIC |
| 6318.018 | 1 | | 0.503 | | 0.110 |  | -129.716 | 263.432 |
|  | 15395 | | 7744 | | 3387 |  |  |  |
| [6296-6340] | [15341-15448] | | [0.488-0.519] | | [0.1-0.12] |  |  |  |
|  |  | |  | | Two_epoch(NSS) |  |  |  |
| Theta | Na | | nu | | T |  | Log likelihood | AIC |
| 8002.329 | 1 | | 0.263 | | 0.106 |  | -128.131 | 260.262 |
|  | 19499 | | 5128 | | 4134 |  |  |  |
| [7818-8186] | [19050-19946] | | [0.246-0.280] | | [0.086-0.126] |  |  |  |
|  |  | |  | | Three_epoch(TS) |  |  |  |
| Theta | Na | nuB | | nuF TB | | TF | Log likelihood | AIC |
| 6501.396 | 1 | 0.566 | | 0.015 0.181 | | 7.53e-05 | -125.380 | 258.76 |
|  | 15842 | 8967 | | 238 5735 | | 2 |  |  |
| [6351-6652] | [15475-16209] | [0.544-0.588] | | [0.01-0.02] [0.114-0.248] | | [4.78e-5-1.028e-4] |  |  |
|  |  |  | | Three_epoch(NSS) | |  |  |  |
| Theta | Na | nuB | | nuF TB | | TF | Log likelihood | AIC |
| 8213.493 | 1 | 0.274 | | 0.057 0.123 | | 0.00017 | -127.296 | 262.592 |
|  | 20013 | 5484 | | 1141 4923 | | 7 |  |  |
| [8081-8345] | [19691-20334] | [0.253-0.295] | | [0.056-0.058] [0.107-0.139] | | [0.00017] |  |  |

NOTE: Performance of three different demographic models (neutral equilibrium model, two-epoch model and three-epoch model) based on polymorphism data for TS and NSS populations. The upper level of parameters is reported with respect to the ancestral population size Na as in ∂a∂i. The lower level of parameters is physical unit transformed as indicated in Materials and Methods
