# Supplementary figures and images for: Fast diffusion of domesticated maize to temperate zones
Source: Sci Rep. 2017 May 18;7:2077. doi: 10.1038/s41598-017-02125-0 (PMC5437101; doi:10.1038/s41598-017-02125-0)

**Supplementary Figure S1**

K=2

K=3

SS NSS mixed TS


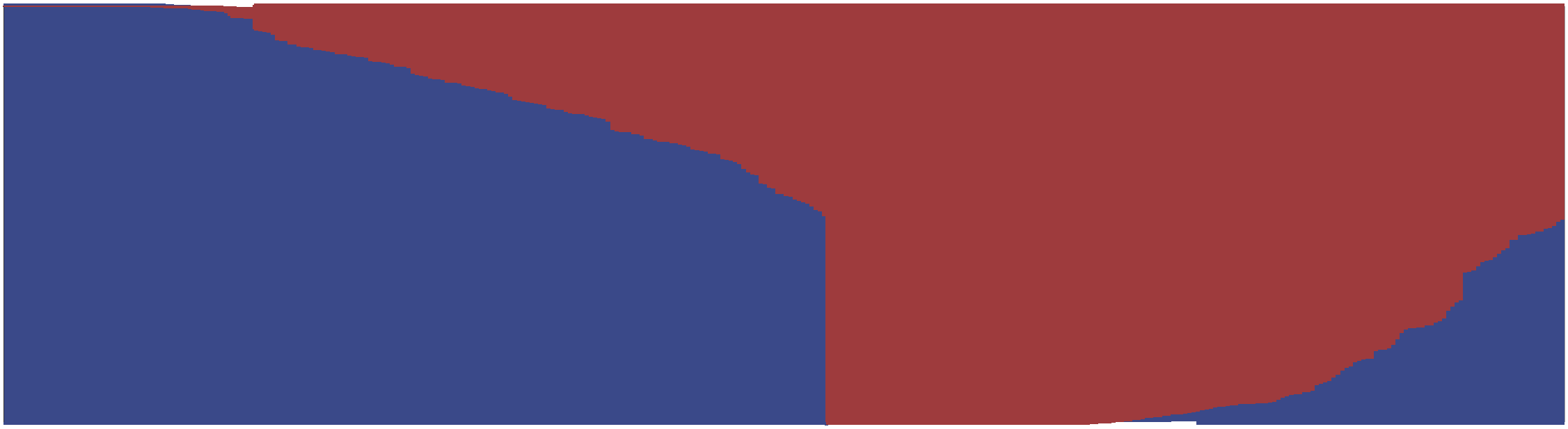

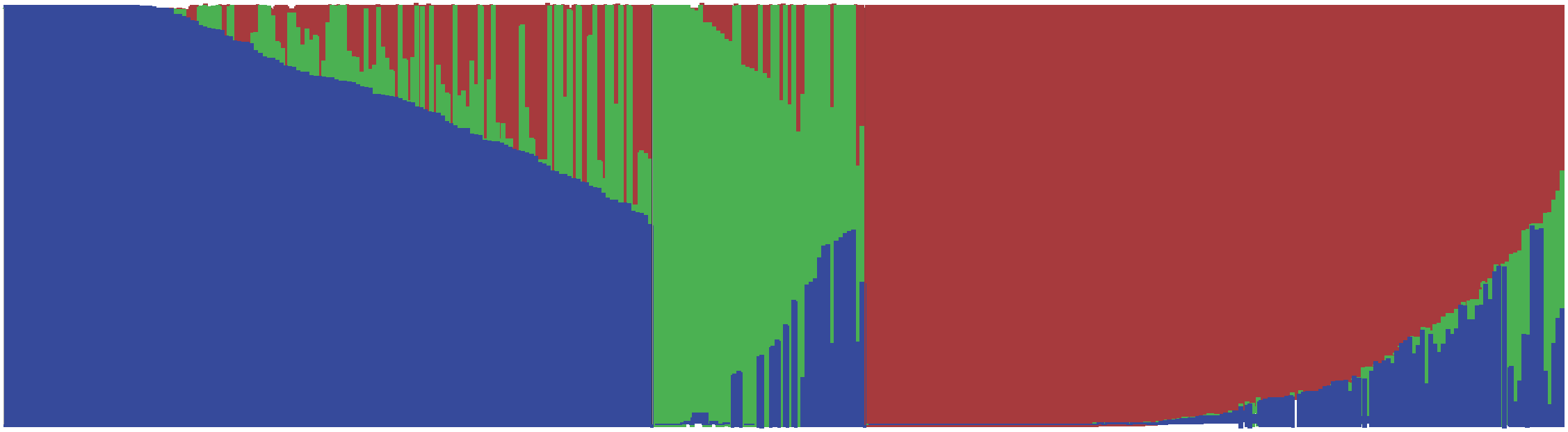

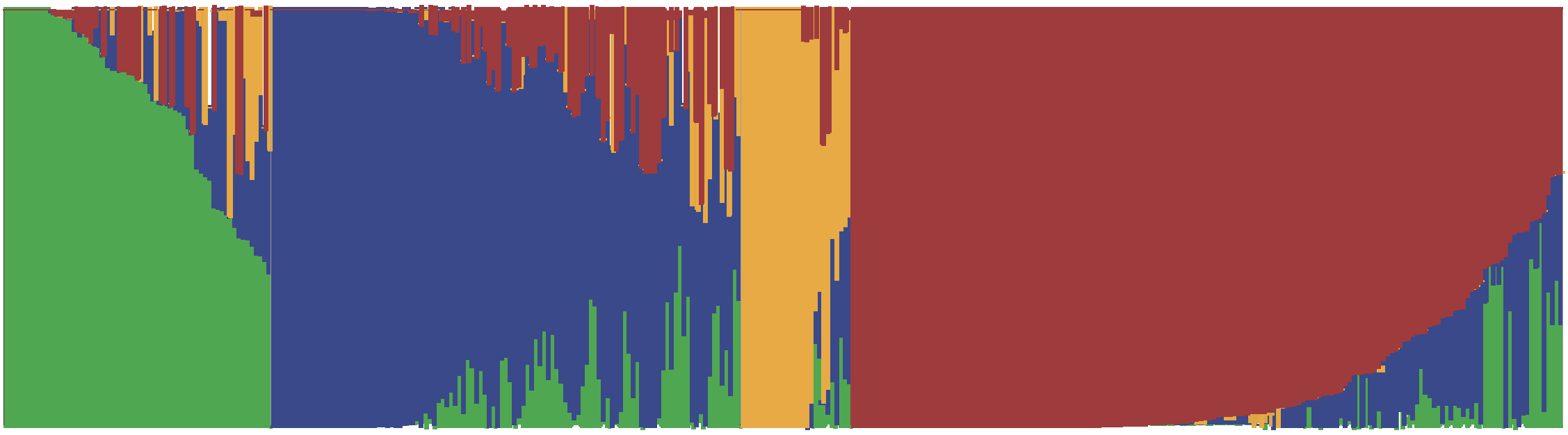


K=4

K=5


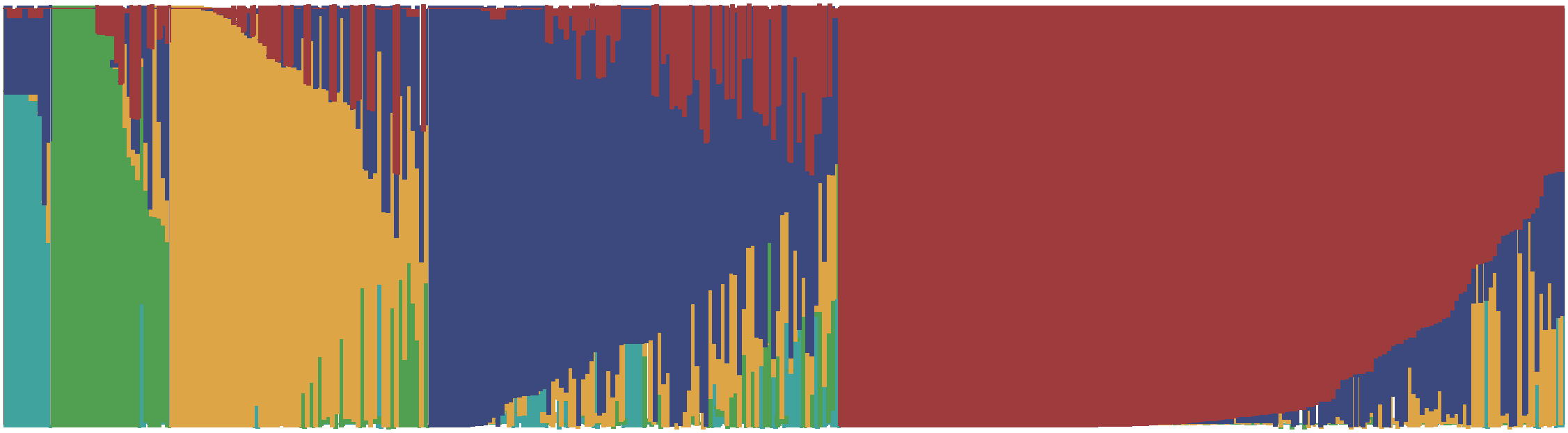


**Supplementary Figure S2**

**
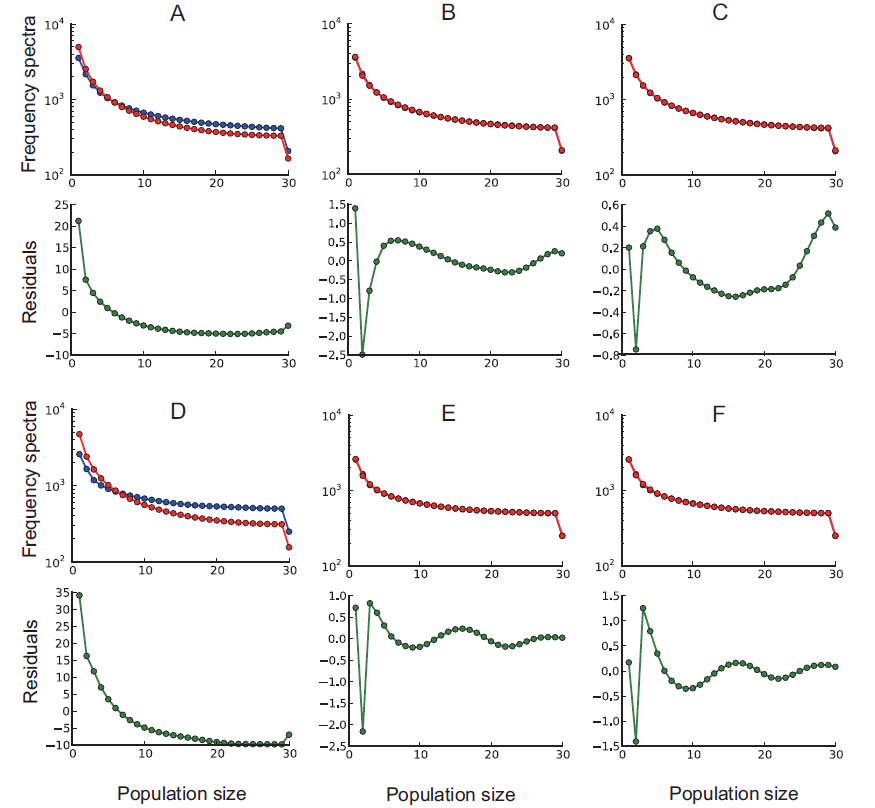
**

**Supplementary Figure S3**


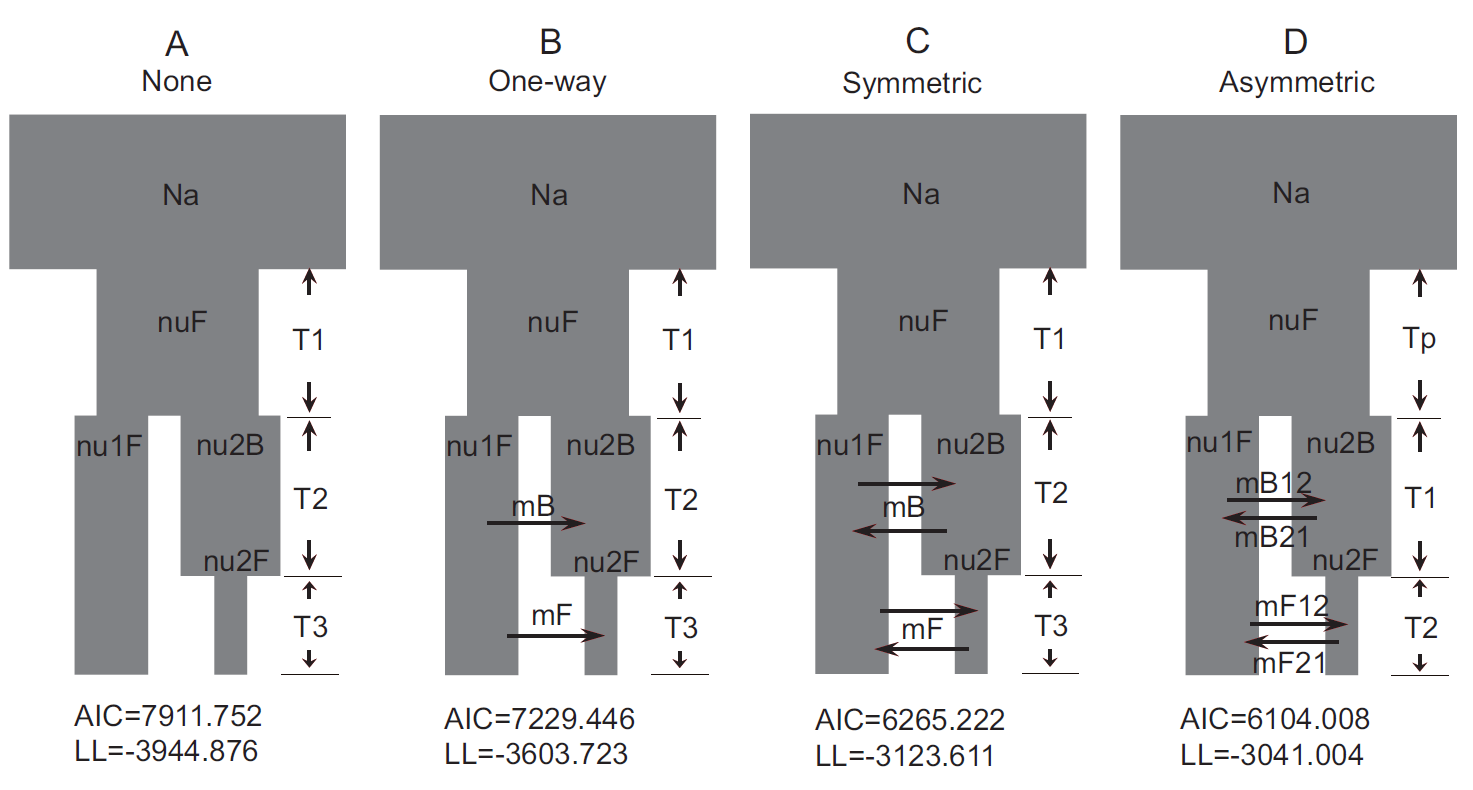

Supplement: Supplementary file 6 — Dataset 5 [file 41598_2017_2125_MOESM6_ESM.doc]
